# Prediction of Lung Function Status Using Handgrip Strength and Anthropometry among the Healthy Malay Population in Malaysia

**DOI:** 10.3390/healthcare11071056

**Published:** 2023-04-06

**Authors:** Mohd Hasni Ja’afar, Rosnah Ismail, Noor Hassim Ismail, Zaleha Md Isa, Azmi Mohd Tamil, Nafiza Mat Nasir, Tengku Saifudin Tengku Ismail, Nurul Hafiza Ab Razak, Najihah Zainol Abidin, MyLinh Duong, Khairul Hazdi Yusof

**Affiliations:** 1 Department of Community Health, Faculty of Medicine, Universiti Kebangsaan Malaysia, Cheras, Kuala Lumpur 56000, Malaysia; drmhasni@ukm.edu.my (M.H.J.);; 2 Department of Primary Care Medicine, Faculty of Medicine, Universiti Teknologi MARA Sungai Buloh, Sungai Buloh 47000, Selangor, Malaysia; 3 KPJ Tawakkal KL Hospital, Jalan Pahang Barat, Kuala Lumpur 53000, Malaysia; 4 Department of Diagnostic & Allied Health Science, Faculty of Health and Life Sciences, Management & Science University, Shah Alam 40100, Selangor, Malaysia; 5 Population Health Research Institute (PHRI), Hamilton Health Sciences and McMaster University, Hamilton, ON L8L 2X2, Canada

**Keywords:** adult, spirometry, lung functions, handgrip strength, Malaysia

## Abstract

Lung function depends primarily on the strength of the intercostal muscles and the diaphragm, which is indirectly related to handgrip strength (HGS). This study aims to determine the predictability of lung functions using HGS among healthy adults of Malay ethnicity in Malaysia. This study also aims to compare the equation using HGS with equations without HGS, such as the Global Lung Initiative (GLI). This study was carried out among adults between 35 to 70 years of age residing in urban and rural Malaysia. A series of standardized questionnaires were used to collect socio-demographic information. Lung functions were measured using a portable spirometer and HGS was measured using a Jamar dynamometer. The predictability of lung function indices (FEV_1_ and FVC) using HGS, age, and height was determined using multiple linear regression (MLR). Prediction of lung function indices was also generated using models without HGS for comparison with the equation that used HGS from this study. Pearson correlation analysis showed that both dominant (r = 0.49; *p* < 0.001) and non-dominant (r = 0.58; *p* < 0.001) HGS had a moderate significant correlation with lung function. In the MLR model, HGS was a significant (*p* < 0.001) predictor of lung function indices (FEV_1_ and FVC). The correlation of the predicted and measured lung indices using the equation generated in this study, which includes HGS, was higher compared with other lung function test equations that do not include HGS. The equations from MLR could be used to predict lung function indices among healthy Malay adults. The measurement of HGS may be used as a screening tool for lung function status when spirometry is unavailable.

## 1. Introduction

Biological indices such as forced vital capacity (FVC) and forced expiratory volume in 1 s (FEV_1_) are usually used to determine a person’s lung function status. FEV_1_ is used to evaluate the severity of respiratory diseases such as asthma, chronic obstructive pulmonary disease (COPD), and interstitial lung disease, while FVC is used to evaluate the overall function of the lungs and to determine the presence of any obstructive or restrictive lung diseases [1,2]. FEV_1_ and FVC are measured together to provide a more comprehensive assessment of lung function and to determine the type and severity of the respiratory disease [2]. The test to collect these measurements requires forceful exhalation of air from the lung and uses a spirometer. However, spirometry is still under-utilized due to its high costs and inadequate training among health professionals [3]. A study conducted in Malaysia concluded that patients’ perception of spirometry tests was that they were difficult and troublesome [4]. In addition, Park et al. determined that respiratory muscle strength is a partial determinant of the success of the lung function test, as it requires the contraction and relaxation of respiratory muscles such as the diaphragm and intercostal muscles, which change intrathoracic expansion and lung volume [5].

Handgrip strength (HGS) is a measurement collected using a dynamometer in which maximal isometric grip force is applied for a short duration [6]. HGS is an acceptable, convenient test that assesses an individual’s overall muscle strength and is a potential indicator of health conditions [6,7]. Studies have reported that decreased HGS is associated with all-cause, cardiovascular, and non-cardiovascular mortality [8]; frailty [6]; chronic obstructive airway diseases; and spine-related problems among the elderly [7]. HGS has also been found to be related to lung function indices such as FVC and FEV1 among healthy populations of adolescents, young adults, and elderly individuals [3,9,10]. Thus, HGS may indirectly indicate overall skeletal muscle strength, including the strength of muscles related to normal lung function [11]. Mgbemena et al. suggested that validation of a consistent relationship between HGS and lung function across a range of populations would support the applicability of HGS as a simple and inexpensive assessment tool [7].

On top of that, Abdullah et al. concluded that the existing global lung function 2012 equation (GLI) was less accurate when applied in an Asian setting [12]. Therefore, this study aims to examine the ability of HGS and anthropometry to predict lung function indices (FEV_1_ and FVC) among the healthy Malay population in Malaysia. This study also aims to compare the equation using HGS with those without HGS such as GLI and other studies conducted in Malaysia.

## 2. Methodology

The design of the PURE study has previously been described elsewhere [13]. In brief, PURE is a large-scale international study of the incidence, mortality, and risk factors associated with non-communicable diseases that include individuals from urban and rural communities in 21 countries, including Malaysia. This study enrolled 15,792 Malaysian adults between the ages of 35 and 70 years at baseline.

Sampling was conducted through community leaders followed by home visits. All eligible individuals living in the same household were invited to volunteer for the study. The feasibility of carrying out long-term follow-up was considered when selecting the study sites and participants. All participants provided written informed consent and the protocol was approved by the Hamilton Health Sciences Research Ethics Board (PHRI), the Research and Ethics Committee of the UKM Medical Centre and the Research Ethics Committee of the UiTM (Project code: PHUM-2012-01).

To ensure standardized methods of data collection, research assistants were trained with comprehensive operation manuals, videos, and workshops. Data were transferred electronically to the project office at the Population Health Research Institute (PHRI) for quality control checks of the data.

### 2.1. Procedures

Trained research assistants administered standardized, validated questionnaires to extract self-reported demographic information such as gender, age (from date of birth), ethnicity (Malay and non-Malay), education level (none, primary, secondary or tertiary), occupation (blue collar, white collar, or homemaker), marital status (single, married, or separated), socio-economic status (low, middle, or high), location (urban or rural), smoking status, and history of cardiovascular diseases (CVD). Only participants with complete questionnaire data, HGS, and acceptable and reproducible lung function data were included in this study. Those with a history of CVD (hypertension, stroke, angina, and heart failure) and respiratory diseases (COPD, asthma, and tuberculosis) who were considered in the PURE study were excluded in this study. Further exclusion criteria were those with a history of diabetes mellitus and active smokers.

Spirometry measurements were performed using a portable spirometer device (MicroGP, MicroMedical, Chatham, IL, USA) due to its affordability and ease of use with an accuracy of ±2% [14]. A disposable mouthpiece was used for each participant. Each participant attempted up to six forced expiratory maneuvers while standing with a straight back and wearing a nose clip. Measurements of maximum effort and forced exhalation for at least 6 s were taken. The three highest measurements of FEV_1_ and FVC were recorded. This method followed the American Thoracic Society guidelines for lung function tests [15]. Spirometer calibration with a 3L syringe was performed monthly and when deemed necessary by local staff. For analyses in this study, we selected participants with at least three measurements of FEV_1_ and FVC with maximum effort, without cough, and within 150 mL variability for analyses [14,15].

HGS was measured using a Jamar dynamometer (Sammons Preston, Bolingbrook, IL, USA) according to a standardized protocol described previously [16]. The participant’s arm was positioned vertically to the body and the dynamometer was held with the elbow flexed to 90°. The participant was instructed to squeeze the device as hard as possible for 3 s. The measurement was repeated thrice, with intervals of at least 30 s between measurements. Three measurements were taken from each hand of each participant. In this study, the maximum values obtained from each hand were used (referred to as dominant HGS and non-dominant HGS). Height and weight were measured using a portable height measuring scale stature meter and a TANITA (BC-558 Ironman^®^) segmental body composition analyzer. Participants’ height without shoes on was measured to the nearest centimeter (cm) using a portable height measuring scale stature meter. Weight was measured to the nearest kilogram (kg) using TANITA (BC-558 Ironman^®^) segmental body composition analyzer.

### 2.2. Statistical Analysis

Data analysis was conducted using IBM SPSS version 26.0. Anthropometric characteristics of the participants were presented as means and standard deviation (SD), for normally distributed data. Pearson’s correlation was computed to assess the correlation between the HGS and lung function indices. T-tests and ANOVA were used to compare lung function indices across groups of different ages, gender, marital status, education level, occupation, socioeconomic status (SES), and communities.

Multiple linear regression (MLR) was used to model HGS, age, and height by gender group to measure FEV_1_ and FVC. Other potential independent predictor variables (marital status, occupation, SES, location, and education level) were excluded in the regression due to multicollinearity and interaction with gender and age. Prediction of the lung function indices was also generated using a model suggested by GLI, TMC, and Singh et al. [12,17,18]. Then, the Spearman correlation test was done to determine the correlation between the measured and predicted lung function indices. Assumptions of linearity, independence of errors, homoscedasticity, and normality of residuals were met. All of the statistical tests used a two-tailed comparison with a 95% level of confidence. A total of 3112 participants completed the lung function indices and HGS measurements, and did not have missing values for age, gender, and height.

## 3. Results

The mean HGS values for this study population were 24.33 (±8.75) kg and 22.19 (±8.37) kg for dominant and non-dominant hands, respectively (Table 1). The mean lung function indices for this study population were 1.87 (±0.57) L for FEV_1_ and 2.07 (±0.66) L for FVC. The mean age of this study population was 49.39 years (±8.84 years) and the mean height was 155.68 (±8.04) cm.

Pearson correlation analysis showed that dominant HGS had a statistically significant moderate positive relationship with FEV_1_ (r = 0.54; *p* < 0.001) and FVC (r = 0.52; *p* < 0.001). Similarly, Table 2 shows there were significant positive relationships between non-dominant HGS and FEV_1_ (r = 0.51; *p* < 0.001) and FVC (r = 0.49; *p* < 0.001).

Table 3 shows the sociodemographic characteristics of age, marital status, education level, occupation type, SES, and location. There was a decreasing trend in lung function indices as the age of respondents increased. The differences were significant for FEV_1_ (F = 97.07; *p* < 0.001) and FVC (F = 72.52; *p* < 0.001). Those who worked in blue- or white-collar jobs had a trend toward higher lung function indices than their counterparts who were homemakers. The differences were also significant for FEV_1_ (F = 199.11; *p* < 0.001) and FVC (F = 181.48; *p* < 0.001). There was an increase in lung function indices observed as SES increased. The differences between groups were significant for FEV_1_ (F = 47.97; *p* < 0.001) and FVC (F = 54.50; *p* < 0.001).

The MLR analysis produced an equation that was significantly able to be used as a predictor of lung function indices. The study generated the following regression equations proposed for predicting the lung function indices:

For prediction using dominant HGS (DHGS):

$$\begin{array}{c} {\mathrm{FEV}}_{1\;}\left(\mathrm{male}\right)=0.014\;\left(\mathrm{DHGS}\right)+0.024\;\left(\mathrm{HEIGHT}\right)-0.019\;\left(\mathrm{AGE}\right)-1.107\\ {\mathrm{FEV}}_{1\;}\left(\mathrm{female}\right)=0.010\;\left(\mathrm{DHGS}\right)+0.017\;\left(\mathrm{HEIGHT}\right)-0.017\;\left(\mathrm{AGE}\right)-0.269\\ \mathrm{FVC}\;\left(\mathrm{male}\right)=0.019\;\left(\mathrm{DHGS}\right)+0.025\;\left(\mathrm{HEIGHT}\right)-0.017\;\left(\mathrm{AGE}\right)-1.314\\ \mathrm{FVC}\;\left(\mathrm{female}\right)=0.014\;\left(\mathrm{DHGS}\right)+0.018\;\left(\mathrm{HEIGHT}\right)-0.016\;\left(\mathrm{AGE}\right)-0.292 \end{array}$$

For prediction using non-dominant HGS (NDHGS):

$$\begin{array}{c} {\mathrm{FEV}}_{1}\left(\mathrm{male}\right)=0.011\;\left(\mathrm{NDHGS}\right)+0.026\left(\mathrm{HEIGHT}\right)-0.020\;\left(\mathrm{AGE}\right)-1.194\\ {\mathrm{FEV}}_{1}\left(\mathrm{female}\right)=0.009\;\left(\mathrm{NDHGS}\right)+0.017\left(\mathrm{HEIGHT}\right)-0.017\;\left(\mathrm{AGE}\right)-0.269\\ \mathrm{FVC}\;\left(\mathrm{male}\right)=0.015\;\left(\mathrm{NDHGS}\right)+0.027\;\left(\mathrm{HEIGHT}\right)-0.019\;\left(\mathrm{AGE}\right)-1.410\\ \mathrm{FVC}\;\left(\mathrm{female}\right)=0.013\;\left(\mathrm{NDHGS}\right)+0.018\;\left(\mathrm{HEIGHT}\right)-0.017\;\left(\mathrm{AGE}\right)-0.299 \end{array}$$

The predicted lung function indices were slightly higher than the measured lung function indices, except for the FVC of males (Table 4). The correlation coefficients demonstrate that there are significant moderate correlations between the measured and predicted values of FEV_1_ (male: DHGS, r = 0.572; NDHGS, r = 0.560; and female: DHGS, r = 0.501; NDHGS, r = 0.498; *p* < 0.001). Similar correlations were shown for FVC (male: DHGS, r = 0.528; NDHGS, r = 0.514; and female: DHGS, r = 0.445; NDHGS, r = 0.441; *p* < 0.001). The correlation of predicted and measured lung indices using the equation generated in this study was higher compared with other equations that do not use HGS. For instance, the correlation of predicted and measured FEV_1_ among male using DHGS was r = 0.572 (*p* < 0.001), while Singh et al., TMC, and GLI showed a correlation of r = 0.544, r = 0.532 and r = 0.532 (*p* < 0.001), respectively. A similar trend of correlation was observed for the other lung indices.

## 4. Discussion

This study was carried out to examine the ability of HGS and anthropometry to predict lung function indices (FEV_1_ and FVC) among the healthy Malay population in Malaysia. Additionally, this study aimed to compare the equation using HGS with those without HGS, such as GLI and other studies conducted in Malaysia. We found HGS was a significant predictor along with traditional lung function indices predictors such as height and age. The equation suggested in this study showed a better correlation between predicted and measured lung function indices than previous equations proposed by GLI, TMC, and Singh et al. [12,17,18]. To the best of our knowledge, this study is the first attempt to integrate HGS in the lung function equation involving data from a large healthy population residing in both urban and rural areas in Malaysia, and then compare it with traditional equations that only include height and age.

The mean values of both DHGS and NDHGS were found to be higher in males than those in females among this study population, which is consistent with previous findings [3,12,19,20,21]. The reason for the findings is that males have a longer head-tuberosity length of the radius bone, therefore longer flexor and extensor muscle of the forearm compared with females, which allowed for greater muscle contractile units of the hands, causing higher power DHGS and NDHGS [7,22,23]. The findings of the study, which indicate a statistically significant difference in FEV_1_ and FVC between males and females, were consistent with prior research in this area [7,12,20,21]. Males were expected to have higher lung function indices compared with females because they tend to be taller and have a wider chest, which resulted in a larger lung size [3,24]. In fact, lung function indices (FEV_1_ and FVC) are proportional to body size, which means that a taller person will have a larger intrathoracic space, allowing for higher lung expansion and volume compared with a shorter person [3,25]. Moreover, Bellemare et al. stated that the difference between lung volumes among genders was approximately 10–12% higher in males than in females who had the same height and age [26]. This study also indicates a statistically significant decreasing trend of FEV_1_ and FVC with increasing age, which is consistent with previous studies [21,27,28,29]. According to Lee et al., FEV_1_ decreased by approximately 20 mL/year at age ranges from 25 to 39 years old and the rate will be progressively increased to 35 mL/year after the age of 65 years old [30]. Previous studies have also reported that aging is associated with sarcopenia, defined as a reduction in skeletal muscle mass [27,29,31]. Skeletal muscle mass is related to respiratory muscle strength, wherein sarcopenia could affect the pulmonary function [29,31]. Bahat et al. concluded that the impairment of pulmonary function due to the weakening of respiratory muscles can cause ineffective cough, which explains the reduced FEV_1_ and FVC values the among elder population [27].

Both dominant and non-dominant HGS were shown to be significant moderately correlated with the lung function indices (FEV_1_ and FVC) of the participants. These results are similar to previous findings [3,19,20,21]. This study found that DHGS had a statistically significant moderate positive relationship with FEV_1_ (r = 0.54; *p* < 0.001) and FVC (r = 0.52; *p* < 0.001), which were comparable with the results reported by Mgbemena et al. (FEV_1_ (r = 0.64; *p* < 0.0005) and FVC (r = 0.61; *p* < 0.0005)), Chen et al. (FEV_1_ (r = 0.65; *p* < 0.001) and FVC (r = 0.69; *p* < 0.001)) and Zhu et al. (FEV_1_ (r = 0.75; *p* < 0.001)) [3,20,21]. Similarly, there were significant positive relationships between NDHGS and FEV_1_ (r = 0.51; *p* < 0.001) and FVC (r = 0.49; *p* < 0.001) in this study, which were comparable with the results reported by Mgbemena et al. (FEV_1_ (r = 0.63; *p* < 0.0005) and FVC (r = 0.61; *p* < 0.0005)) [3].

On top of that, MLR in this study showed that both dominant and non-dominant HGS are significant predictors of lung function indices (FEV_1_ and FVC) after being adjusted for age and height. This finding was similar to studies done in China and Nigeria [3,20,21]. Mgbemena et al. reported that both DHGS and NDHGS were significant predictors of FEV_1_ and FVC when gender, height, age, and weight were adjusted [3]. Both studies done by Zhu et al. and Chen et al. found that DHGS was a significant predictor of FEV_1_ and FVC after being adjusted for other factors [20,21].

These findings could be explained by the strong relationship reported between skeletal muscle strength and respiratory muscle strength (maximal inspiratory pressure (MIP) of the diaphragm and maximal expiratory pressure (MEP) of expiratory muscle strength) [3,32]. A study done in Turkey that involved 62 male nursing home residents showred that HGS is significantly related to MIP and MEP (r = 0.35; *p* < 0.01 and r = 0.26; *p* < 0.05, respectively) [27]. Then, regression analysis of the study revealed that only MIP is significantly related to HGS when age, BMI, and history of the cerebrovascular accident were controlled [27]. Meanwhile, a study done in Korea among 65 healthy elderly reported that HGS is significantly related to MIP and MEP (r = 0.560; *p* < 0.01 and r = 0.393; *p* < 0.05, respectively) [32]. Both studies showed that MIP has a higher correlation value with HGS compared with MEP, which might be explained by the decrease in respiratory muscle strength that may be affected by MIP earlier than MEP among the geriatric population [27,32]. Bahat et al. suggested that a reduced MIP translates to lower lung function indices in an individual and could suggest an impairment in the lungs [27]. The moderate correlation between HGS and lung function indices reported in this study could be an indicator of a healthy state of the participants’ respiratory systems.

Furthermore, a previous study showed that FEV_1_ and FVC could mediate the decrease in mobility controlled by a decline in muscle strength and power [33]. HGS has also been used as a muscle strength indicator, which is evaluated together with pulmonary function parameters of FEV_1_ and FVC [19]. Other than that, a previous study by PURE reported that the prognostic value of HGS was proven to be independently associated with all-cause mortality, cardiovascular mortality, and cardiovascular disease when other factors such as dietary habits, physical activity levels, and socioeconomic status were controlled [8]. Therefore, a reduction in HGS is likely to indicate reduced lung function.

Moreover, compared with other equations that were not included HGS, such as GLI, TMC, and Singh et al., the equation using HGS generated in this study had a better correlation between predicted and measured lung function indices [12,17,18]. This study found the correlation of predicted and measured FEV_1_ among female using DHGS and NDHGS were r = 0.501 and r = 0.498 (*p* < 0.001), respectively. Meanwhile, the equation from Singh et al., TMC, and GLI showed a correlation of r = 0.486, r = 0.478, and r = 0.478 (*p* < 0.001), respectively [12,17,18]. Notably, a similar trend of correlation was observed for the prediction of FEV_1_ among males and FVC for both genders. This result indicates that the addition of HGS in the existing equation, which only included height and age, improved the prediction of the lung function indices. This is because HGS is associated with lung function indices, particularly FEV_1_ and FVC, which has been demonstrated in this study and previous studies [3,19,20,21].

The main strength of this study is that it was the first attempt to integrate HGS in the lung function equation involving data from a large healthy population of both urban and rural areas in Malaysia. The limitation of this study was the cross-sectional study design that only included baseline data. Thus, the causal and temporal effect of the relationship between HGS and lung indices were not considered. Another limitation of this study is that only Malay ethnicity was included, in which the equations using HGS generated in this study may only be applied to this population in Malaysia. However, Hossain et al. concluded that HGS of Malaysian population was not related to ethnicity, but more related to age and anthropometric data [34]. Apart from that, this study also did not account for other factors influencing the lung function (e.g., thoracic cage deformities and interstitial lung diseases) and only included healthy adults based on the participants’ self-reported data. Further study with a prospective design and comparison between healthy and unhealthy lung function participants would warrant more insight regarding the relationship of HGS and lung function indices.

## 5. Conclusions

In conclusion, HGS is a useful alternative screening method of lung function status among healthy adults of Malay ethnicity in Malaysia. This study showed that lung function indices were predicted by HGS in MLR. Furthermore, compared with other equations that do not include HGS, the equation using HGS generated in this study had a better correlation between predicted and measured lung indices. The lung function indices (FEV_1_ and FVC) that were predicted by the equation from this study portrayed healthy lung function indices rather than unhealthy lung function, which could be used as a guideline for a healthy predicted FEV_1_ and FVC value. Thus, the equation from the MLR could be used to predict lung function status during rehabilitation or in general use by a health practitioner. It would be useful to measure HGS as a screening tool to detect lung function status in clinics with no access to spirometry, such as those in remote areas. Well-designed prospective studies on HGS are needed to understand its association with lung function and may contribute to establishing the importance of HGS in pulmonary health screening and rehabilitation.

## Figures and Tables

**Table 1:** Characteristics of the respondents.

Variables		Mean (±S.D)		
		Overall *(N = 2901)*	Male *(N = 785)*	Female *(N = 2116)*
Age (year)		49.39 (8.84)	51.21 (8.94)	48.71 (8.71)
HGS (kg)				
	Dominant	24.33 (8.75)	33.40 (8.97)	20.97 (5.76)
	Non-dominant	22.19 (8.37)	30.96 (8.45)	18.93 (5.52)
Lung function indices				
	FEV_1_ (L) *	1.87 (0.57)	2.31 (0.62)	1.70 (0.46)
	FVC (L) *	2.07 (0.66)	2.55 (0.72)	1.89 (0.54)
Physical measurement				
	Height (cm)	155.68 (8.04)	163.30 (7.01)	152.86 (6.41)
	Weight (kg)	64.32 (13.55)	69.28 (13.38)	62.49 (13.15)

* FEV_1_ = forced expiratory volume in 1 s; FVC = forced vital capacity.

**Table 2:** The correlation coefficient between lung function indices and HGS.

Variables	Correlation Coefficient (r)	
	FEV_1_	FVC
HGS		
Dominant	0.54 *	0.52 *
Non-dominant	0.51 *	0.49 *

* *p*-value <0.001.

**Table 3:** Comparisons of lung function indices by respondents’ characteristics.

Variable	N	FEV_1_ (L) Mean (±SD)	Test	FVC (L), Mean (±SD)	Test
Age (year)			F = 97.07; *p* < 0.001 *		F = 72.52; *p* < 0.001 *
35–40	535	2.03 (0.56)		2.22 (0.67)	
41–50	1152	1.98 (0.56)		2.19 (0.65)	
51–60	834	1.77 (0.53)		2.00 (0.62)	
61–70	380	1.50 (0.53)		1.68 (0.59)	
Marital status			F = 31.08; *p* < 0.001 *		F = 29.39; *p* < 0.001 *
Single	76	1.95 (0.73)		2.13 (0.73)	
Married	2595	1.89 (0.57)		2.10 (0.66)	
Separated	218	1.57 (0.52)		1.74 (0.61)	
Education level			F = 87.90; *p* < 0.001 *		F = 78.74; *p* < 0.001 *
None	264	1.55 (0.53)		1.73 (0.58)	
Primary	764	1.70 (0.55)		1.89 (0.63)	
Secondary	1322	1.93 (0.54)		2.14 (0.63)	
Tertiary	551	2.09 (0.58)		2.32 (0.67)	
Occupation			F = 199.11; *p* < 0.001 *		F = 181.48; *p* < 0.001 *
White collar	1003	2.04 (0.58)		2.27 (0.67)	
Blue collar	568	2.05 (0.62)		2.26 (0.70)	
Homemaker	1291	1.64 (0.47)		1.82 (0.54)	
SES status			F = 47.97; *p* < 0.001 *		F = 54.50; *p* < 0.001 *
Low	811	1.79 (0.61)		2.00 (0.71)	
Medium	1945	1.96 (0.59)		2.18 (0.69)	
High	356	2.14 (0.52)		2.45 (0.65)	
Gender			t = 25.02; *p* < 0.001 *		t = 23.18; *p* < 0.001 *
Male	785	2.31 (0.61)		2.55 (0.72)	
Female	2116	1.70 (0.46)		1.89 (0.54)	
Location			t = 6.35; *p* < 0.001 *		t = 4.42; *p* < 0.001 *
Rural	1402	1.80 (0.57)		2.02 (0.68)	
Urban	1499	1.93 (0.57)		2.12 (0.64)	

* significant at *p* < 0.001.

**Table 4:** Comparison between the measured and predicted values of the lung function indices.

	FEV_1_ (L)		FVC (L)	
Mean (±S.D)	Male *(N = 785)*	Female *(N = 2116)*	Male *(N= 785)*	Female *(N = 2116)*
Measured	2.31 (0.62)	1.70 (0.46)	2.55 (0.72)	1.89 (0.54)
Predicted using HGS				
DHGS ^a^	2.31 (0.35)	1.71 (0.23)	2.53 (0.38)	1.97 (0.24)
NDHGS ^b^	2.37 (0.34)	1.67 (0.22)	2.49 (0.37)	1.87 (0.24)
Predicted without HGS				
Singh et al. [18] ^c^	2.37 (0.42)	1.66 (0.31)	2.79 (0.44)	2.03 (0.31)
TMC ^d^	2.13 (0.34)	1.65 (0.23)	2.44 (0.36)	1.83 (0.23)
GLI ^e^	3.24 (0.36)	2.65 (0.23)	3.44 (0.36)	2.83 (0.23)
Correlation coefficient (r)				
DHGS	0.572 *	0.501 *	0.528 *	0.445 *
NDHGS	0.560 *	0.498 *	0.514 *	0.441 *
Singh et al. [18]	0.544 *	0.486 *	0.490 *	0.422 *
TMC	0.532 *	0.478 *	0.477 *	0.415 *
GLI	0.532 *	0.478 *	0.477 *	0.415 *

* significant at *p* < 0.001 (2-tailed); ^a^ lung indices = b_1_ × DHGS + b_2_ × H + b_3_ × A + b; ^b^ lung indices = b_1_ × NDHGS + b_2_ × H + b_3_ × A + b; ^c^ lung indices = b_1_ × H + b_2_ × A + b; ^d^ log lung indices = b_1_ × logH + b_2_ × logA + b; ^e^ log lung indices = b_1_ × logH + b_2_ × logA + b + 1. DHGS = dominant hand grip strength; NDHGS = non-dominant hand grip strength; H = height; A = age; TMC = The Malaysian Cohort; GLI= Global Lung Initiative.

## Data Availability

The data presented in this study are available upon request from the corresponding author.

## Abbreviations

HGS: handgrip strength; DHGS: dominant handgrip strength; NDHGS: non-dominant handgrip strength; FEV_1_: forced expiratory volume in 1 s; FVC: forced vital capacity; GLI: Global Lung Initiative; TMC: The Malaysian Cohort; PURE: Prospective Urban and Rural Epidemiological Study; CVD: cardiovascular diseases; SES: socio-economic status; MLR: multiple linear regression.
